# Metallization and Biopatterning on Ultra-Flexible Substrates via Dextran Sacrificial Layers

**DOI:** 10.1371/journal.pone.0106091

**Published:** 2014-08-25

**Authors:** Peter Tseng, Ivan Pushkarsky, Dino Di Carlo

**Affiliations:** Department of Bioengineering, University of California Los Angeles, Los Angeles, California, United States of America; Texas A&M University, United States of America

## Abstract

Micro-patterning tools adopted from the semiconductor industry have mostly been optimized to pattern features onto rigid silicon and glass substrates, however, recently the need to pattern on soft substrates has been identified in simulating cellular environments or developing flexible biosensors. We present a simple method of introducing a variety of patterned materials and structures into ultra-flexible polydimethylsiloxane (PDMS) layers (elastic moduli down to 3 kPa) utilizing water-soluble dextran sacrificial thin films. Dextran films provided a stable template for photolithography, metal deposition, particle adsorption, and protein stamping. These materials and structures (including dextran itself) were then readily transferrable to an elastomer surface following PDMS (10 to 70∶1 base to crosslinker ratios) curing over the patterned dextran layer and after sacrificial etch of the dextran in water. We demonstrate that this simple and straightforward approach can controllably manipulate surface wetting and protein adsorption characteristics of PDMS, covalently link protein patterns for stable cell patterning, generate composite structures of epoxy or particles for study of cell mechanical response, and stably integrate certain metals with use of vinyl molecular adhesives. This method is compatible over the complete moduli range of PDMS, and potentially generalizable over a host of additional micro- and nano-structures and materials.

## Introduction

Polydimethylsiloxane (PDMS) forms the base of a large proportion of microdevices, and has seen extensive use in microfluidics [1], [2], flexible electronics [3]–[8], and in developing cell-material interfaces [9]–[15]. A large proportion of traditional devices are fabricated using the standard formulation, a 10∶1 ratio of polymer base to crosslinker that possesses an elastic modulus of approximately 2 MPa.

Ultra-flexible formulations of PDMS, which can be straightforwardly generated through increasing the base to cross-linker ratio up to 70∶1, can typically achieve elastic moduli down below 3 kPa [14] which could have unique advantages for flexible electronics and as cell biology substrates. These flexible substrates, however, are relatively underutilized, particularly in terms of surface micromachining and in integration of these surfaces with complex microstructure. PDMS at this flexibility is unique to stiffer formulations in that they deflect under significantly lower stresses than standard PDMS, and their viscoelasticity lends a minor self-healing quality to the layers. This could potentially yield a new avenue for flexible electronics, which often utilize composite structures of plastics and membranes of 10∶1 PDMS. These ultra-flexible substrates have found the most use in cell biology, as PDMS moduli can approximate the moduli of tissues at ratios of 70 to 50∶1 base to crosslinker ratios. At the elastic moduli created with these formulations, cells can significantly deflect the substrate on their own, without macroscopic stimuli. These substrates are diversely utilized for traction force microscopy [12], [13], [16] (measuring deflections generated by cells), stem cell differentiation [17], studying cell polarization [9], in which the goal is often to assay how stiffness of the substrata can affect cellular behavior [18].

In general, however, surface micromachining or patterning of PDMS at these extremely soft formulations is not straightforward due to complications in manipulating this layer. The main issue stems from the fact that PDMS at these formulations is typically tacky, non-specifically adheres over a wide variety of surfaces, and is generally difficult to pattern [19]. For example, siloxanes designed for this elastic modulus (such as Sylgard 527) are commonly used as adhesives. Standard methods of lithographically patterning standard PDMS [20] (such as with SU-8) are incompatible with soft formulations of PDMS due to layer incompatibilities with solvents, and large stresses that form during processing. Oxygen plasma exposure, often used to improve adhesion to stiffer formulations of PDMS, is similarly not directly amenable to patterning on ultra soft layers due to the formation of brittle, easily cracked oxide monolayers [21], [22]. Direct contact printing approaches similarly lead to poor pattern transfer due to deformation of the underlying PDMS substrate, and nonspecific interactions between stamps and the substrate [19]. Microstructure is commonly embedded in PDMS through physical demolding of PDMS from silicon substrates [23]–[25]. The same issues with stamping are encountered in demolding, as nonspecific interactions and the weak physical nature of soft PDMS often leads to significant deformation or destruction of the elastomer layer.

Water-soluble sacrificial layers have previously been studied as a method of releasing microstructure in surface micromachining [26]. These possess a number of advantages over traditional sacrificial layers, such as solvent or gas (ie. XeF2) based methods, namely their convenience in deposition and preparation (spin-coating, and low temperature baking), and the broad compatibility of the aqueous release step. This release step also potentially makes this compatible with a number of ultra soft (elastic modulus <30 kPa) hydrogels and polymers.

Polyvinyl alcohol has seen initial work as either an intermediate, transfer carrier [27] for delicate structure fabricated on one substrate to another, or in transferring protein patterns onto PDMS and acrylamide hydrogels [19]. Despite its durability (it is stable as a free membrane), we found direct printing on these materials to be difficult due to poor adhesion of microstructure to native layers.

**Figure 1:**
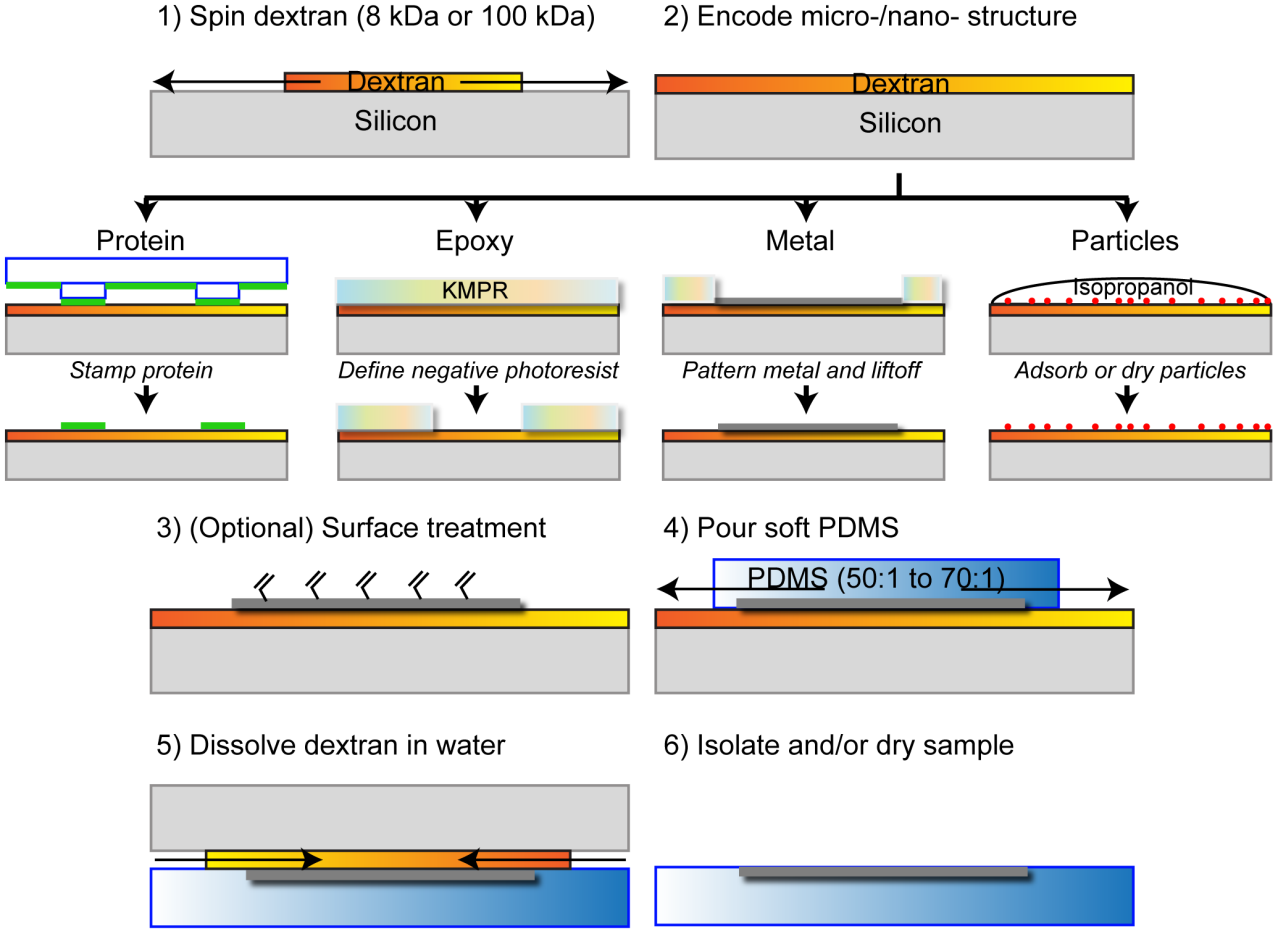
Process flow for dextran-based surface micromachining of ultra-soft PDMS (50 to 70∶1). Dextran is spun and dried on silicon pieces, before deposition of various micro- and nano-structures. PDMS is subsequently cross-linked directly above these substrates, micromachined PDMS is released in water.

In this work, we utilize water-soluble dextran thin films coated on rigid silicon wafers as a direct template for the stable lithographical patterning and deposition/adsorption of micro- and nano-scale features (Fig. 1). We found dextran, with proper surface treatment, to be a stable and durable host for these complex microstructures. Features patterned by this method are treated (if required), and directly crosslinked and/or embedded within ultra-flexible PDMS (50 to 70∶1) during its crosslinking step, in contrast with previous approaches. Samples can be subsequently detached by sacrificial etching of dextran in water. In particular, this approach allows potentially destructive steps (including plasma treatments, thick film lithography processing) to take place on dextran instead of ultra-flexible PDMS layers, yielding clean, composite structures of PDMS and micropatterned materials. For example, plasma-treated dextran layers were also an excellent substrate for contact printing proteins, which transferred readily with minimal applied weight and contact time. Dextran layers are compatible with solvent-based lithographical processing of negative photoresists, for example developers such as polypropylene glycol monomethyl ether acetate, solvent rinses (acetone, methanol, isopropanol), and solvent-based photoresist strippers (n-methyl pyrrolidinone) [26], which do not lead to dextran dissolution, unlike aqueous stripping solutions.

Notably, the wide range of chemical moieties that can be conjugated to the dextran used as the sacrificial layer for PDMS substrates allows tuning of wetting/surface adsorption characteristics of the elastomer beyond those of standard PDMS. We found that this approach stably integrated epoxy, proteins, metals, and particles into PDMS of variable stiffness, and we utilized composite structures generated by this method to pattern cells, measure deflections induced by cells, and study cell self-patterning in dual material systems.

## Results and Discussion

### 1. Transfer of dextrans and proteins into PDMS

We utilized the direct crosslinking of PDMS above dextran sacrificial substrates to integrate both dextran and proteins into PDMS. Strong bonding between dextran/protein and the PDMS network is encouraged by a number of interactions, including 1) a strong molding effect due to the high mobility of PDMS monomers during the initial prepolymerization stage of the interaction, and 2) covalent bonding between proteins and the PDMS through a so-called “poisoning” of the catalyst used in Sylgard 184 formulations of PDMS by various protein side chains, namely amino- and thiol- bearing amino acids [28], [29], yielding covalent bonds between the siloxane network and the proteins. These effects help stabilize the introduction of dextran polymers (directly from the thin film layer), and patterned protein into the PDMS matrix. Once PDMS films were cured at room temperature (typically over 5 to 7 days), the dextran thin films were sacrificed directly in deionized water. Samples could finally be dried gently under a stream of pressurized air.

**Figure 2:**
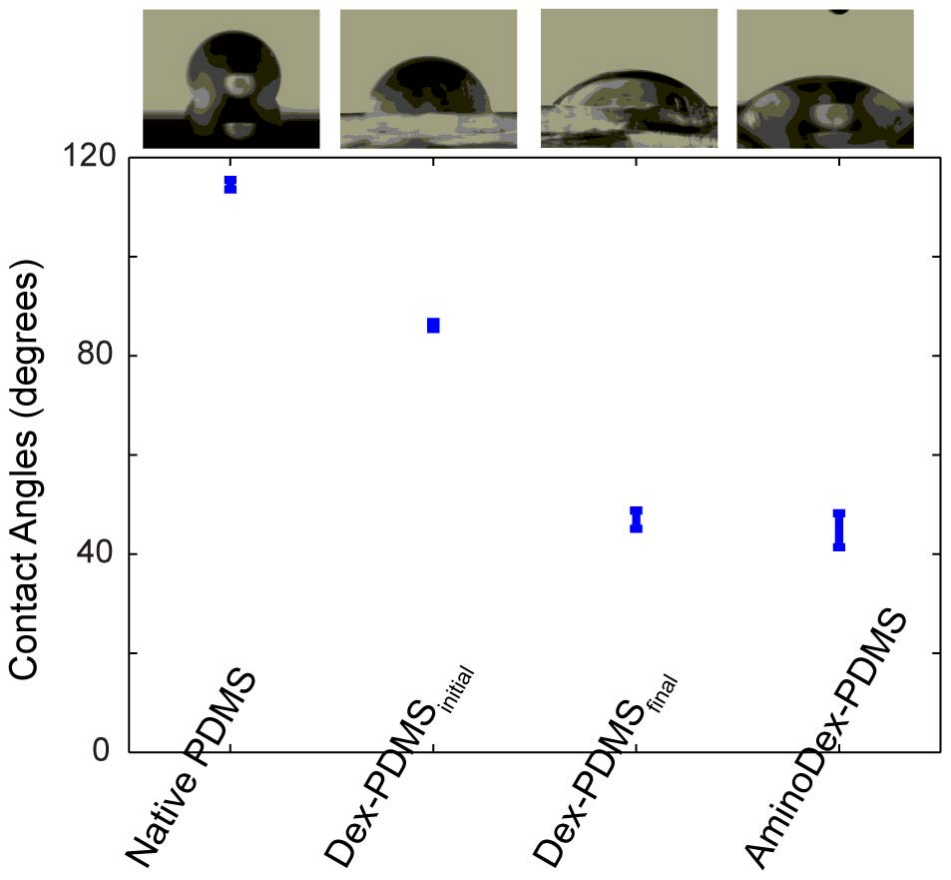
Contact angle of various formulations of dextran-PDMS. Introduction of dextran onto the PDMS surface increases the wetting of the substrate, depending on formulation of PDMS, and absorption of water into the embedded dextran.

Dextran introduced onto PDMS surfaces by this method shifted both wetting and surface adsorption characteristics of the substrate. For example, introduction of simple dextran polymers onto PDMS slightly reduced initial contact angles (from 110 degrees to 90 degrees), but also significantly increased the retention of water onto these substrates (Fig. 2). This is likely due to the high water absorbing nature of dextran polymers. This could be clearly quantified by agitating water droplets, as contact angles uniformly dropped to 40 degrees after shock. We similarly investigated the introduction of amino bearing dextran polymers into PDMS, and their effect on wetting characteristics. Surfaces embedded with amino-dextran possessed initial contact angles of 40 degrees without need of agitation (Fig. 2). More valuably, dextran-mediated surface shifts were stable, and these substrates responded similarly at 1 day and 30 day exposure to air, conditions that would inevitably result in hydrophobic recovery of surface-treated PDMS.

**Figure 3:**
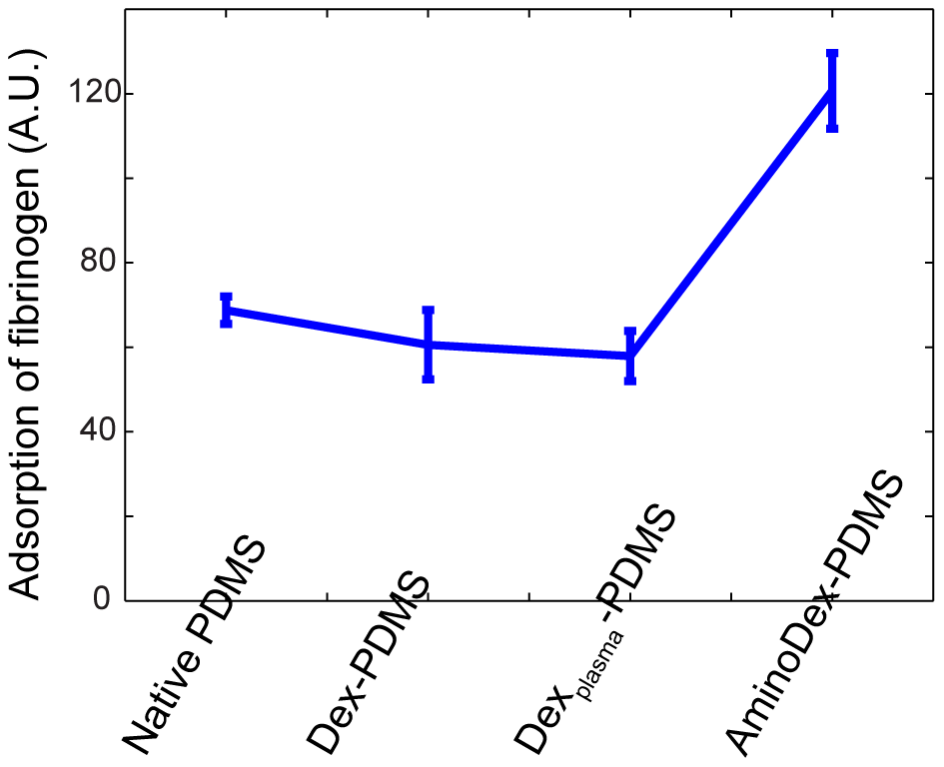
Adsorption characteristics of various formulations of dextran-PDMS (60∶1). Unmodified dextran-embedded PDMS possesses similar adsorption to native PDMS, while amino-modified dextrans heavily adsorb fibrinogen.

The protein adsorption characteristics of dextran films can vary significantly with the deposition process [30], [31]. Interestingly, the presence of unconjugated dextran polymer on PDMS (both untreated and plasma-treated) had little effect on the adsorption of fibrinogen, which we assayed by measuring fluorescent intensity of stabilized protein. Introduction of amino-dextran, however, significantly increased the adsorption of fibrinogen, possibly due to electrostatic interactions between the negatively charged fibrinogen, and positively charged amino-dextran (Fig. 3). Conjugated dextrans are fairly common and commercially available, and our results suggest that dextran embedding may be an alternative method of stably modifying the surface characteristics of PDMS and introducing stable functional groups.

**Figure 4:**
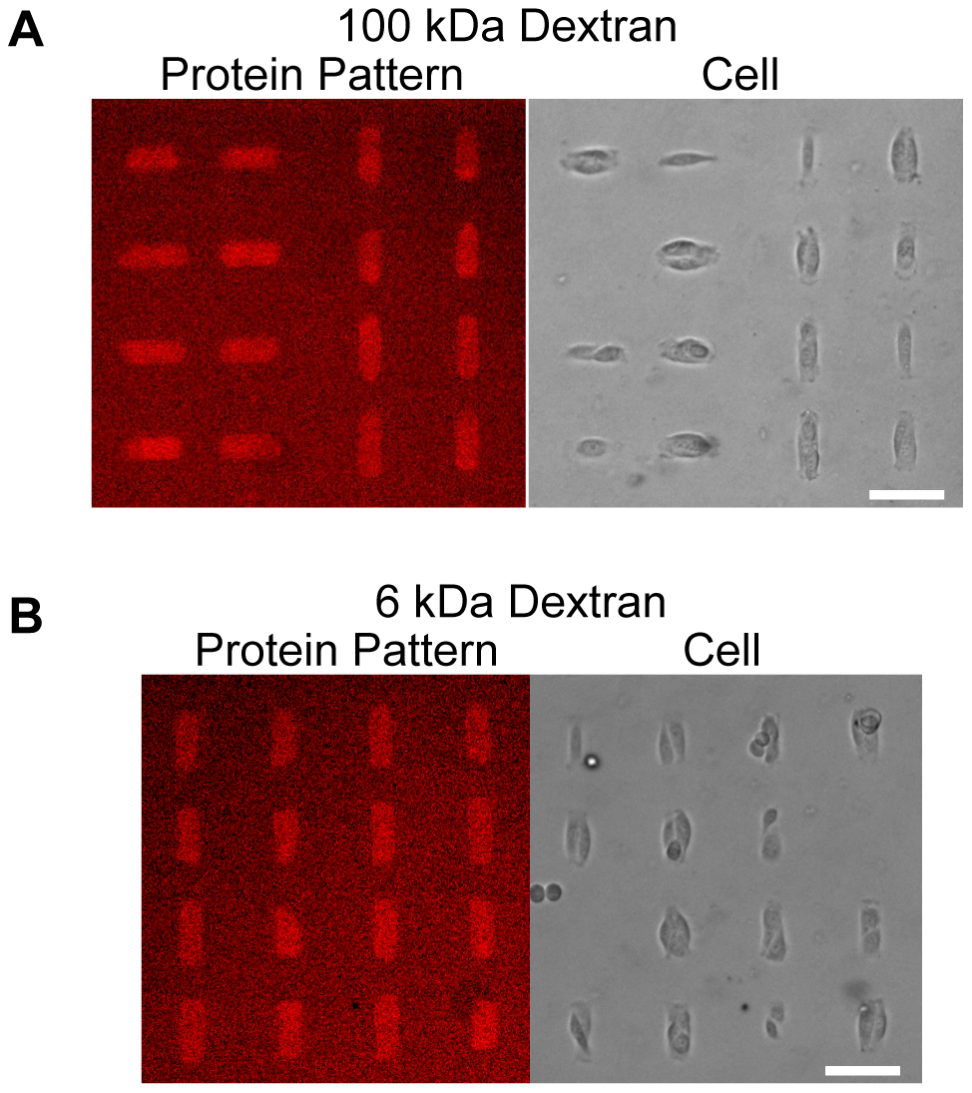
Cell patterning. **a) and b) Live-cell images of cells cleanly patterned on fibronectin islets following 2 days of culture for both 100 kDa and 6 kDa dextran formulations.** Scale bar is 60 µm.

Fibronectin could further be integrated into PDMS via micro-contact printing on top of dextran thin films prior to PDMS curing over the entire assembled surface (Fig. 4). A short plasma treatment of the dextran thin film (5 to 10 seconds) significantly improved the ease and fidelity of this step, although it was not explicitly necessary. Proteins robustly transferred during the PDMS (50 to 70∶1) crosslinking, likely aided by the poisoning and crosslinking to the PDMS via poisoning of the crosslinker as discussed earlier. This provides a noticeable advantage over previous work with PVA, as an intermediary sulfo-SANPAH conjugation step was required for proper stabilization of the protein pattern. This approach could be presumably extended to a variety of contact printed proteins.

We tested the fidelity of these patterns over up to 5 days, with the use of several molecular weights of dextran, both 100 kDa and 6 kDa. On both these formulations, cells localized over the extents of fibronectin patterns during the time frames of the experiment suggesting that PDMS oligomers did not seep beneath and cover the patterned protein. Protein pattern transfer and reproducibility was >99%, as determined by the frequency of defects, such as aberrant points of fluorescent signal, appearing in patterns (Figure S1). Individual protein patterns stamped over .5 cm transferred with high reproducibility down to at least 8 µm. As protein patterns are separated via a contact-less method, the fidelity of the pattern reproduction is directly dependent on the stamping protocol. We ran preliminary assays on cell viability and growth on dex-PDMS substrates (Figure S2). Cells grown on 10∶1 dex-PDMS, 60∶1 dex-PDMS, and 60∶1 PDMS had similar cell viabilities on day 1 (93.7% ±0.72 on 10∶1 dex-PDMS, 92.8% ±0.93 on 60∶1 dex-PDMS and 93.2% ±1.0 on 60∶1 PDMS) and day 3 (92.6% ±0.5 on 10∶1 dex-PDMS, 93.5% ±1.2 on 60∶1 dex-PDMS, and 92.6% ±0.4 on 60∶1 PDMS), and cells proliferated at similar rates (approximately 2.5-fold, 2.4-fold and 2.3-fold growth on 10∶1 dex-PDMS, 60∶1 dex-PDMS and 60∶1 PDMS samples, respectively, p>>0.05).

### 2. Integrating epoxy layers into soft PDMS

**Figure 5:**
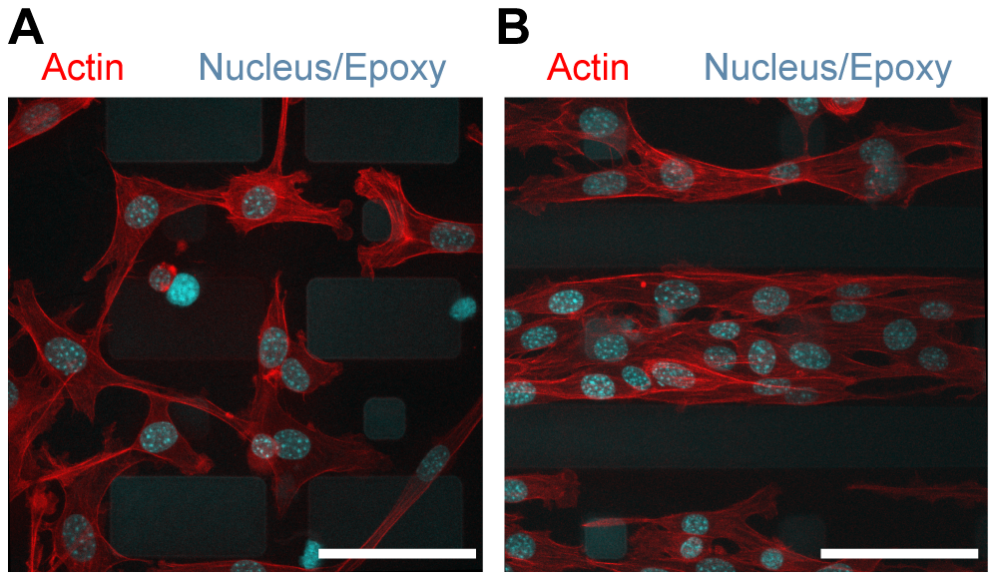
Cell self-patterning above epoxy-PDMS substrates. a) and b) Cells self-patterning on epoxy-PDMS. Cells strongly prefer PDMS portions of the substrate due to preferential adsorption of protein onto these regions, and perhaps develop adhesions towards “stiff” regions adjacent to epoxy. Scale bar is 60 µm.

We also were able to integrate more complex substructure into soft PDMS layers, with the purpose of studying cell response to composite material structures (Fig. 5). Negative photoresists provided a simple target material, as these can be patterned directly via photolithography, and whose processing has been previously demonstrated on dextran thin films. We patterned KMPR photoresist of 40 µm height, and subsequently treated the surface to encourage a stable epoxy-PDMS bond. We tried several silanization steps, including allyl- and amino- silanes, and ultimately found a hydrolytically stable APTES-based approach yielded the best results [32]. This method was composed of a plasma-activation, vacuum deposition of the silane, and subsequent annealing in a humid environment, leading to a nucleophilic surface for subsequent bonding to siloxane. PDMS was finally poured over these substrates and allowed to cross-link at room temperature, before release in phosphate-buffered saline solution.

To study cell response on these composite structures, we incubated substrates in a mixture of fibronectin and fluorescent fibrinogen, and finally seeded 3t3 cells onto these substrates. Interestingly, we found that cells displayed clear self-patterning on these composite structures, strongly preferring the PDMS layer over the epoxy layer. The origin of this seems due to protein adsorption heavily favouring the PDMS over the KMPR epoxy region, leading cells to exclusively grow above the soft PDMS.

Interestingly, our composite epoxy-PDMS structures likely also possesses stiffness gradients [18] in addition to polarity in protein adsorption, which can potentially be a tool to study the cell behaviour on co-patterns on stiffness and matrix. We believe that the ability to integrate materials (of different mechanical and chemical composition) as well as to shift the surface properties of the PDMS creates a versatile platform for cell study.

### 3. Embedding particles in PDMS surfaces

**Figure 6:**
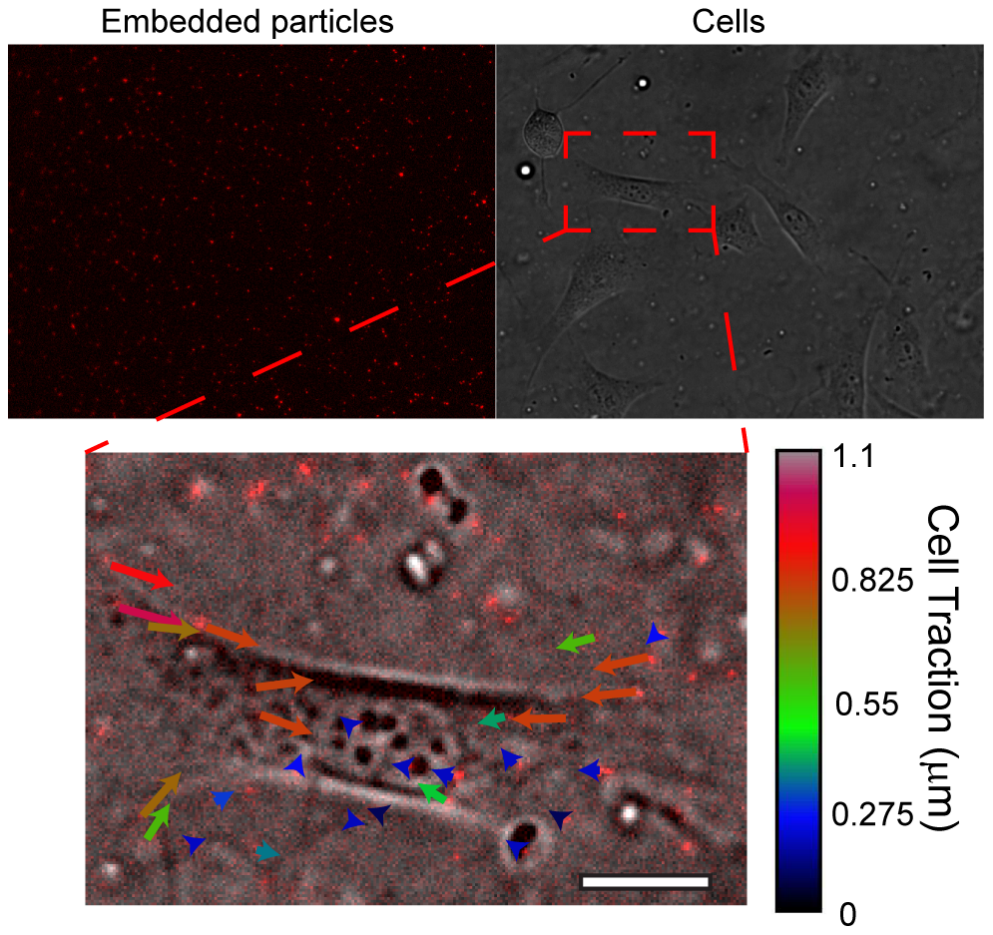
Embedding fluorescent particles directly in PDMS. Particle displacements generated by a single cell. Scale bar is 20 µm.

We additionally assessed the ability of this sacrificial dextran approach to integrate nanoparticles into soft-PDMS substrate surfaces for the purposes of extracting deflections generated by cells during their growth and contraction of their environment (Fig. 6). For hydrogels, surface integration of particles is generally achieved during a demolding process, however, this is not a consistent process for ultra-flexible PDMS due to the strong adhesive forces, as discussed previously. Instead, researchers adsorb APTES onto the PDMS surface, and subsequently bind particles to PDMS surfaces through EDC-NHS reaction chemistry [13], [16]. We reasoned that simple adsorption of particles onto dextran and the embedding of these directly into PDMS would provide a stable substrate for cell deflection studies, without use of reaction chemistries.

Many particles used in biological studies are designed for aqueous stability, and require solvent switching before being compatible with dextran substrates. We pelleted and dried silica particles (sicastar-red), and subsequently resuspended these particles (via aggressive agitation) in isopropanol. When pipetted above dextran substrates, these particles adsorbed readily on to the surface within several minutes. Samples could subsequently be dried under pressurized air, before PDMS processing and crosslinking.

Cultured cells seeded above PDMS (60∶1) with embedded particles generated noticeable deflections in the substrate (up to 1 µm, tracked via particle displacement) when compared to when the cells were subsequently released from the substrate via trypsinization. We did not notice loss of particles during this step, indicating that cells did not internalize or remove the particles from the PDMS surface.

### 4. Embedding metal into ultra-soft PDMS

**Figure 7:**
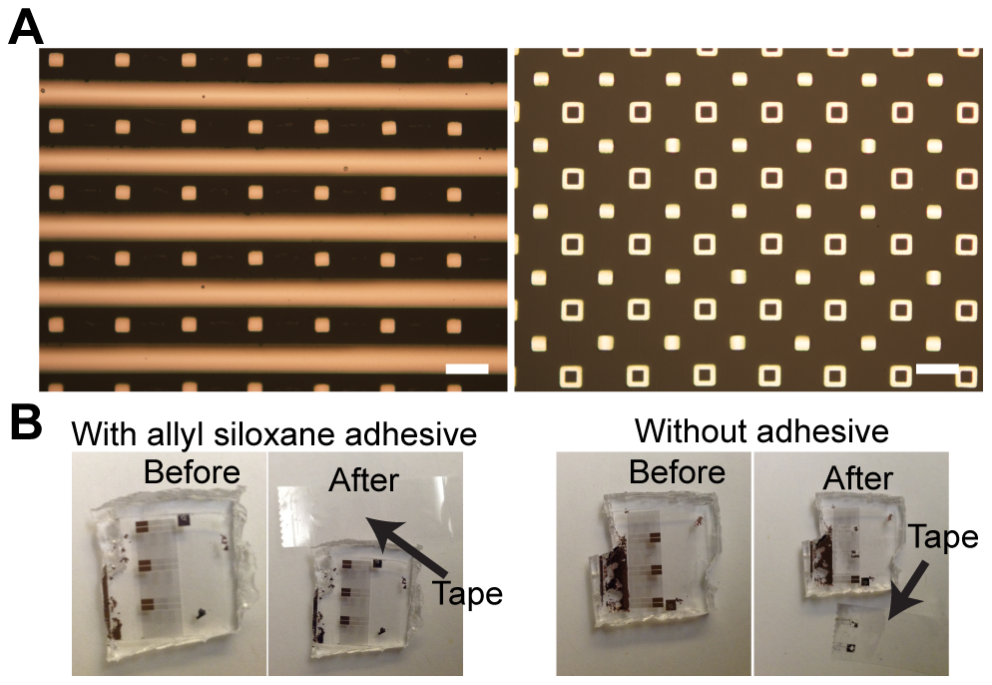
Dextran-mediated transferring of microstructured metal onto PDMS composed of lithographically patterning metal, and silanization by allyltriethoxysilane. a) Thin film metal features (lines and elements, 200 nm thick) of Titanium/Copper/Titanium efficiently transferred onto ultra soft PDMS (60∶1). b) Scotch tape test verifying adhesive capabilities of allyl- based silanes on PDMS (10∶1). Scale bar is 60 µm.

We finally investigated dextran sacrificial layers as a method of introducing patterned, thin film metals onto soft-PDMS substrates (Fig. 7). Metal-PDMS interactions are typically relatively weak, and commonly require plasmas to improve adhesion between materials. The deposition process (either sputtering or e-beam evaporation) also typically generates additional stress on the material, commonly inducing stiffness gradients and wrinkling of the substrate surface [33]–[35]. By transferring the potential stresses generated by these approaches onto dextran thin films, the weakly cross-linked PDMS could remain relatively undamaged.

We patterned metal onto dextran using both e-beam evaporation and sputtering. Adhesion of metals to dextran during e-beam evaporation could be greatly improved through pre-exposure of the substrate to air plasma, and metal microstructures deposited in this fashion remained stable through subsequent KMPR stripping in Remover PG. Sputtered metals typically had lower yields during this step, that varied with thin film thickness and agitation during the KMPR stripping.

We initially identified a molecular adhesive that would operate over a wide range of different metals. While the introduction of gold electrodes on top of fully cross-linked PDMS via mercaptosilanes has been extensively studied [24], this approach is not explicitly compatible with other metals due to the lack of native thiol reactivity in many of the other common micromachined metals (i.e. titanium, copper, chromium, or aluminium). Vinyl- reactive silanes, which interact directly with Sylgard 184 crosslinker presumably offers an alternative molecular adhesive for metals that form a stable oxide layer. We tested the adhesion of titanium-based layers to PDMS (10∶1) using this approach with a scotch-tape test, and found significant improvement in adhesion with the presence of the vinyl siloxane adhesive. We similarly tested this approach with thin film aluminum, however adhesion improvement for these layers was more mild, potentially due to differences in stability in the silanization of respective native oxides.

With this molecular adhesive, we sought to integrate large scale patterns of varying shape and sizes into ultra-flexible PDMS (60∶1). Both long metal lines (TiCuTi, 200 nm thick, e-beam evaporated), and small micron scale features cleanly transferred (with ∼100% efficiency, with or without silane, Fig. 7c) over the extent of the substrate, with no noticeable defects and minimal wrinkling of the thin film, particularly surprising due to the discrepancy in elastic modulus between metal (100 GPa), and the PDMS (<10 kPa). This is likely due to low residual stresses in a room temperature, e-beam evaporative process. Sputtered aluminum thin films, which had mixed improvement in adhesion as discussed previously, nevertheless transferred cleanly onto PDMS substrates. These structures, however, developed slight arching along small single features (<50 µm), and displayed low frequency wrinkling over larger features (>1 mm), likely due to higher residual stress during deposition of these layers [36].

## Conclusions

In this work, we have detailed the use of dextran thin films as a sacrificial template for complex micropatterned features and microstructures, and subsequently utilized these to perform surface micromachining on ultra-soft PDMS (50 to 70∶1 base to crosslinker). Dextran served to diversely generate patterns of epoxy, metal and protein, and as a substrate for adsorption of particles. By crosslinking PDMS above these substrates, and subsequently removing dextran with water, these structures stably integrated onto PDMS surfaces. This could be further stabilized by the use of molecular adhesives, such as APTES, and allyltriethoxysilane. To demonstrate the usefulness of this technique, we demonstrated proof-of-concepts over a range of applications to stably shift PDMS surface properties, pattern cells, study cell response to composite material structures, and finally to measure deflections induced during cellular contractions.

We believe that this methodology can be expanded to a suite of different nanostructures and materials as a means of surface micromachining both PDMS and other ultra-soft materials. The dual capabilities of shifting surface properties with formulations of dextran, and introducing patterned microstructures could be utilized both in biological studies and in developing flexible electronics, particularly those which require low elasticity (i.e. biomedical applications, or biointerfacing).

## Materials and Methods

### 1. Preparation of dextran thin films

Dextran (100 kDa and 6 kDa, from Sigma Aldrich) were diluted into deionized water at a concentration of 20% w/v, and spun (at 1,500 to 2,000 rpm) onto plasma-activated silicon wafers to agoal thickness of approximately 1 µm. Samples were dried on a hotplate at 150°C, and sectioned into pieces for subsequent microfabrication steps. Amino-dextran (70 kDa, Invitrogen) was diluted at 10% w/v, poured onto silicon substrates, and subsequently dried overnight in a fume hood.

### 2. Preparation of Dex-PDMS and measurement of surface characteristics

PDMS (10∶1 for contact angle measurements, and 60∶1 for adsorption studies) was mixed, degassed, and poured onto dextran (100 kDa) or amine-conjugated dextran substrates, and allowed to crosslink at room temperature over 5 days. Samples were excised, and ultra-soft PDMS (60∶1) slabs were mechanically stabilized by application of a backing glass coverslip. Dextran layers were subsequently dissolved overnight in deionized water, or phosphate-buffered saline to yield Dex-PDMS substrates.

Contact angles were measured by a goniometer. Dex-PDMS exhibited contact angle hysteresis which depended on length of prior exposure to water. Final contact angle was determined following application of moderate shock to the substrate which served to initially wet the substrate, and uniformly reduced the contact angle of droplets.

Protein adsorption studies were conducted with fluorescent fibrinogen (Alexa fluor 568 conjugated, Invitrogen). 25 µg/mL protein was pipetted above substrates, and incubated at room temperature for 30 minutes. Fluorescent intensity was subsequently determined via a Nikon inverted fluorescence microscope.

### 3. Preparation of protein patterned PDMS

Protein stamps were fabricated by patterning KMPR photoresist (Microchem) on silicon (10 µm thick), and subsequently crosslinking and demolding of PDMS. Stamps were inked with a mixture of fibronectin, and fibrinogen-Alexa fluor (10 µg/mL, 30 minutes), and dried with pressurized air. Dextran substrates (100 kDa and 6 kDa) were activated via air plasma (Harrick) for 5 seconds, and immediately stamped with protein for 5 minutes. PDMS (60∶1) was then poured above substrates, and crosslinked at room temperature over at least 5 days before dextran layers were dissolved in PBS overnight, and subsequently blocked in 1% Pluronic F127 (Sigma) for 1 hour.

### 4. Preparation of epoxy-PDMS

KMPR films were spun on and patterned using solvent-based lithography directly on dextran thin films (using SU-8 developer). These epoxy structures were stably linked to PDMS during curing using approaches adapted from the literature used to make PDMS nucleophilic [32]. Aminopropyl triethoxysilane (APTES) was deposited on plasma-treated KMPR via vacuum silanization overnight. The layer was cured at high humidity over 4 hours, before PDMS (60∶1) was poured and cured at room temperature over the structures. Dex-sacrificed samples were incubated with a protein mixture (same as in cell patterning studies) for 1 hour in preparation for cell studies.

### 5. Preparation of metal-PDMS

Titanium, copper, titanium layers (30 nm, 140 nm, 30 nm) were evaporated (CHA) onto patterned, air plasma treated KMPR-dextran substrates. KMPR was subsequently stripped in Remover PG (Microchem) over 30 minutes. Samples were treated in air plasma, and molecular adhesive (allyl triethoxy silane, Sigma) was deposited via overnight vacuum silanization. PDMS-metal composite structures were generated as for protein and epoxy approaches using sacrificial dextran.

### 6. Nanoparticle-embedded PDMS

Silica nanoparticles (500 nm, Sicastar Red, Micromod) were pelleted, dried, and resuspended in isopropanol. Particles were diluted 1000∶1 from their initial concentration, and after extensive agitation (vortex and ultrasonic), were pipetted onto dextran substrates, and allowed to incubate over 3 minutes before being dried under pressurized air. Particles were finally embedded in PDMS during the curing process as in previous sections. Protein was subsequently adsorbed onto these substrates as in previous sections.

### 7. Cell culture

Cells (3t3, ATCC CCL-92) were cultured using standard protocols. Cells were grown in complete medium, trypsinized, seeded onto substrates, and allowed to attach and spread over 1 hour. Samples were subsequently washed in complete medium.

Cells seeded on protein patterned PDMS were imaged using bright-field and fluorescence microscopy over a 4-day time frame.

Cells seeded onto epoxy-PDMS substrates were fixed and stained for phalloidin following 1 day, as in previous protocols [37]. Samples were mounted in Slowfade with DAPI, sealed in a coverlip and nailpolish, before imaging under a Leica SP2 confocal microscope.

### 8. Measuring cell displacements via embedded particles

Measurement of cellular displacement followed approaches introduced in previous work [38], [39]. Fluorescent images of cells that were contracting PDMS embedded with particles were imaged using fluorescence and brightfield microscopy. Samples were subsequently washed in PBS, and trypsinized to release cell attachment from the substrate. Embedded particles were finally re-imaged to acquire the initial position of the particles to extract cell-induced force vectors and magnitudes.

Cell displacements were measured by comparing manipulated particles in comparison to nearby particles not subject to cellular forces.

### 9. Measuring cell viability and proliferation on Dex-PDMS and native PDMS

Dex-PDMS (6 each of 60∶1 and 10∶1) and native PDMS (6 of 60∶1) samples were prepared as described. Fibronectin was adsorbed to each sample. 3T3 cells were cultured using standard protocols, trypsinized, and resuspended at a low concentration (<1000 cells/mL) before being seeded onto the samples. Half of all samples were seeded with 300 µL of cell solution and cultured for 24 hours and the other half were seeded with 200 µL of cell solution and cultured for 72 hours. A live/dead assay (Life Technologies L-3224) was used at each time point to obtain counts of live and dead cells. Viability was determined directly for each sample and the proliferation rate was estimated by calculating the ratio of live cells at 72 hrs to 24 hrs and adjusting for volume differences. Student's t-test was used to confirm there were no significant differences in either proliferation or viability between the three types of substrates.

## Supporting Information

Figure S1 **Repeatability of protein micro-patterning via sacrificial dextran layers.** Two separately prepared protein-patterned PDMS samples (65∶1). The consistency seen in the blown-up patterns demonstrates the robustness and repeatability of this patterning approach. Defects in individual patterns were particularly rare, occurring in <1% of patterns. The proteins used in the shown patterns are equal parts fibronectin and fibrinogen-Alexa Fluor imaged using 2 s exposure time with a 10X objective.
(TIF)

Figure S2 **Cell viability and proliferation on dex-PDMS.** 3T3 cells were cultured on dex-PDMS (60∶1 and 10∶1) and native PDMS (60∶1) for 24 hours and 72 hours. At each time point, live and dead cells were counted. Three experiments were done for each case. (A) Mean percentage cell viability defined as live cells over total cells counted. Cell viability was high (>90%) on all substrates at both 24 hours and 72 hours post-seeding. (B) Representative images of live 3T3 cells on each substrate at 72 hours post-seeding, stained with Calcein AM fluorescent dye. Scale bars are 250 µm. (C) Mean estimated cell proliferation, defined as the total number of live cells at 72 hours divided by the total number of live cells at 24 hours. Since unequal volumes of cell solution were used for seeding the two time points, the calculated ratio was adjusted by multiplication with the ratio of the used volumes (3/2). On each substrate, estimated proliferation was similar as cells underwent approximately one doubling (p>>.05). The error bars indicate standard deviation.
(TIF)
