# Supplementary figures and images for: Metallization and Biopatterning on Ultra-Flexible Substrates via Dextran Sacrificial Layers
Source: PLoS One. 2014 Aug 25;9(8):e106091. doi: 10.1371/journal.pone.0106091 (PMC4143360; doi:10.1371/journal.pone.0106091)

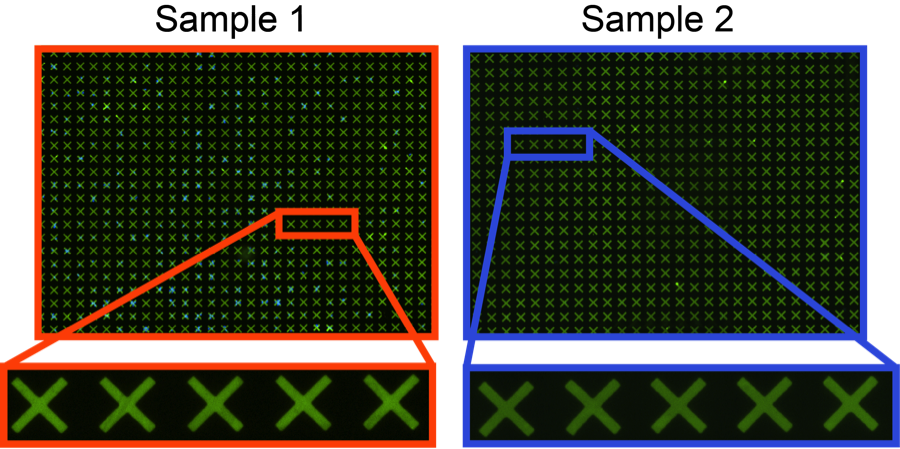

Supplement: Figure S1 — Repeatability of protein micro-patterning via sacrificial dextran layers. Two separately prepared protein-patterned PDMS samples (65∶1). The consistency seen in the blown-up patterns demonstrates the robustness and repeatability of this patterning approach. Defects in individual patterns were particularly rare, occurring in <1% of patterns. The proteins used in the shown patterns are equal parts fibronectin and fibrinogen-Alexa Fluor imaged using 2 s exposure time with a 10X objective. (TIF) [file pone.0106091.s001.tif]

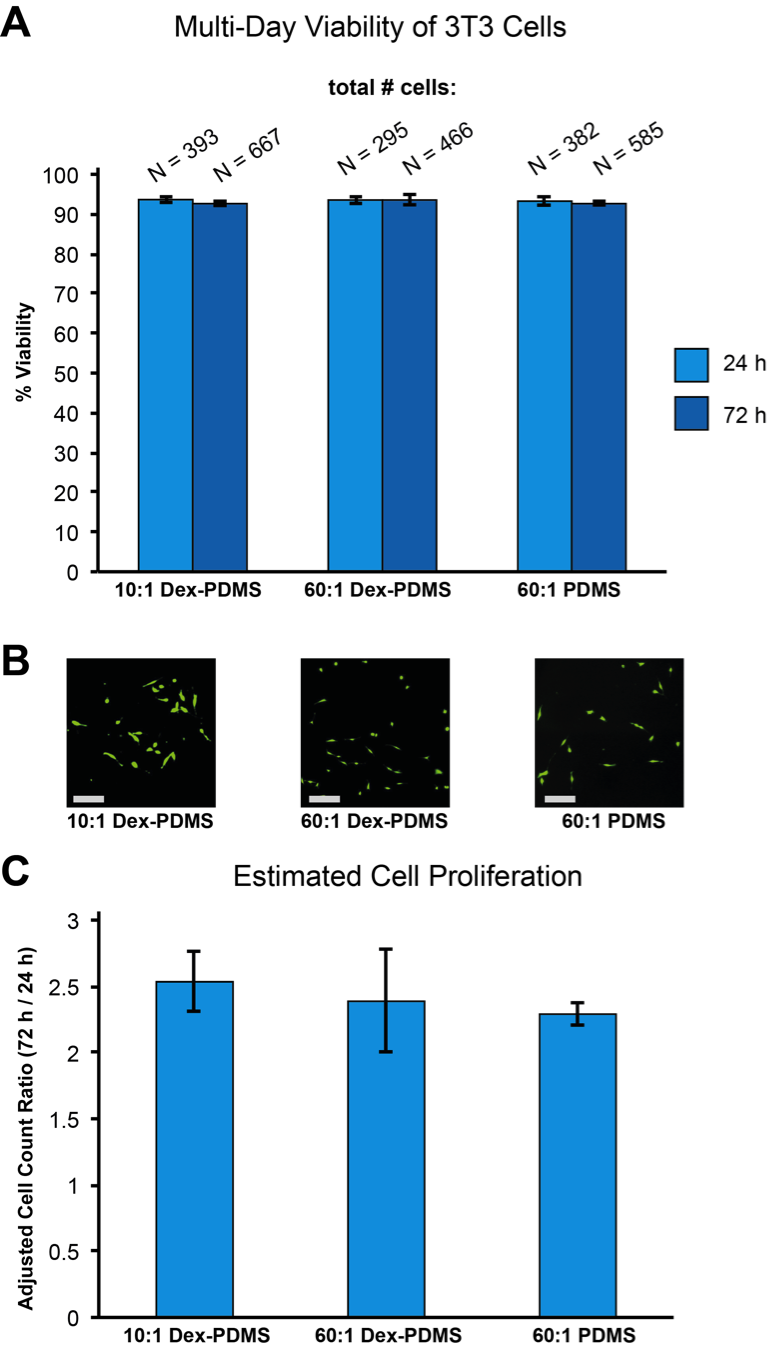

Supplement: Figure S2 — Cell viability and proliferation on dex-PDMS. 3T3 cells were cultured on dex-PDMS (60∶1 and 10∶1) and native PDMS (60∶1) for 24 hours and 72 hours. At each time point, live and dead cells were counted. Three experiments were done for each case. (A) Mean percentage cell viability defined as live cells over total cells counted. Cell viability was high (>90%) on all substrates at both 24 hours and 72 hours post-seeding. (B) Representative images of live 3T3 cells on each substrate at 72 hours post-seeding, stained with Calcein AM fluorescent dye. Scale bars are 250 µm. (C) Mean estimated cell proliferation, defined as the total number of live cells at 72 hours divided by the total number of live cells at 24 hours. Since unequal volumes of cell solution were used for seeding the two time points, the calculated ratio was adjusted by multiplication with the ratio of the used volumes (3/2). On each substrate, estimated proliferation was similar as cells underwent approximately one doubling (p>>.05). The error bars indicate standard deviation. (TIF) [file pone.0106091.s002.tif]
